# Delivery of chemical cargo to endogenous proteins on live cells

**DOI:** 10.3389/fchem.2014.00011

**Published:** 2014-03-12

**Authors:** James J. Chambers

**Affiliations:** Department of Chemistry, Program in Neuroscience and Behavior, University of MassachusettsAmherst, MA, USA

**Keywords:** fluorescent tagging, protein trafficking, fluorescence microscopy, ligand-targeted delivery, traceless labeling

During the past two decades, our collective mastery of gene manipulation, delivery, and fluorescent fusion protein expression has been astounding and has enabled never before dreamed of new lines of research. These advances, coupled with progress in the collection and analysis of microscopy data, have allowed the scientific community to detect proteins in live cells with resolution that was unthinkable not long ago. The confluence of protein engineering and advanced microscopy has enabled us to observe cellular processes at the single molecule level and to begin unraveling the mysteries of cellular biochemistry and dynamics. These advances, however, require delivery of a contrast agent to the subject organism which can sometimes lead to untoward effects and potentially confounded results. Here, I present an opinion on the need to improve and deploy new strategies that allow for the silent tagging of endogenous proteins with contrast agents.

## Where art thou, protein of interest?

Visualization of cells has been enabled by the use of simple organic and inorganic dyes. The results of staining provides the ability to differentiate cell types (e.g., hematoxylin vs. eosin stain) and sometimes offers glimpses of complex cell connectivity (e.g., chromium/silver Golgi stain). In the case of brain tissue, these stains can provide insight into neuronal connectivity (Cajal, 1894). Of course, the utility of these simple dyes and stains is limited in scope to revealing snapshots in time since they require fixation and sometimes development before imaging can be performed.

Arguably, the most difficult challenge facing biology and medicine is the delivery of contrast agents to the brain for the purpose of visualizing the development, plasticity, dysfunction, and degeneration in live neural tissue. The majority of what we know about brain dysfunction comes from tissue snapshots collected from post-mortem samples. These data are robust and offer many insights into biochemical and morphological underpinnings of disease, but the data that these methods can reveal is, by definition, temporally limited to the moment the fixative impregnates the tissue. The detection of changes in cell morphology or, of more relevance to this opinion, responsive dynamic movements of proteins in various subcellular compartments, are lost once the fixative is added.

## Here, protein of interest, can you hold this?

Without question, the field of live cell fluorescence microscopy would be stalled if it weren't for the relatively recent discovery and exploit of natural fluorescent proteins and the boon in molecular biology and genetic delivery strategies (Giepmans et al., 2006). At first, it was the now famous green fluorescent protein from Aequorea victoria. Once the genetic code for GFP was sequenced and packaged into an expression vector, the genie was out of the bottle. One would be hard-pressed now to find any lab that works on questions in cell biology that does not have at least one plasmid containing some form of a boutique GFP variant.

In parallel to the explosion in popularity of GFP and variants thereof, there continues to be the development of new, organic based fluorescent molecules (Mutze et al., 2012). Just when it seems that chemists have exhausted the diversity of chemical and structural space to produce new fluorophores, new ones come along with different properties. These new fluorophores offer combinations of fundamental photophysical properties; some are long-lasting, some are bright (high quantum yield), and some have large Stokes shifts while others photobleach easily, are dim, and excitation and emission wavelengths are not well-separated. However, the old adage, in adapted form, holds here: One molecule's flaw is another molecule's feature. For instance, fast photobleaching chromophores, if photo-reversible, are vital to single molecule imaging techniques used in stochastic optical reconstruction microscopy (STORM imaging) in which molecules are bleached and then a small subset is stochastically turned back on to reveal their sub-diffraction position in space. It is clear that the menu of fluorophores is akin to selecting a fine wine; to make an informed decision, one must understand and appreciate the nuances of the available options.

## How do we deliver these contrast agents to the protein of interest?

The two main methods in use today to deliver fluorescent chromophores to protein targets of interest are to use genetic fusion modification of the target itself or to employ a non-covalent delivery vehicle such as an antibody that has been raised to recognize a specific hapten on the target molecule. Both of these methods are logistically trivial and are routine in many cell biology laboratories.

With currently available commercial technology, generation of a fusion protein containing the target of interest is simple and there are myriad transfection formulations available to deliver the generated plasmid to even the most difficult to transfect cell types (in addition, of course, there are virally-mediated delivery and transgenic strategies). The range of colors for protein-based fluorophores is seemingly infinite, allowing multiplexed experiments (Giepmans et al., 2006) as well as genetically encoded, photoactivatable proteins for single particle tracking of parts of organelles (Manley et al., 2008).

Antibody based delivery of contrast agents has been used extensively in cell biology studies. With advances in synthetic chromophores and new methods for the covalent attachment to antibodies, delivery of these chromophores to targets on live cell surfaces is also becoming routine. The exquisite specificity of the antibody method, coupled with the diversity of commercially available sources for pre-stained antibodies, makes this method very attractive when embarking on a new study.

**Figure 1:**
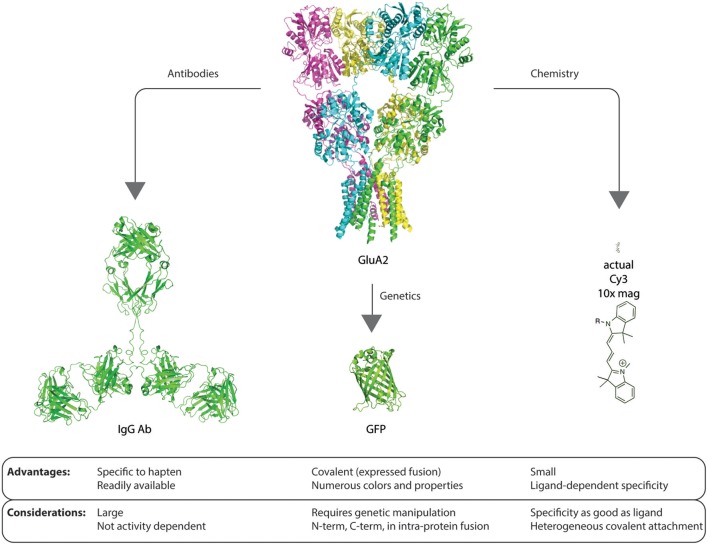
**Graphic example of advantages and considerations of each method of contrast agent delivery to proteins of interest.** The size scale of the glutamate receptor GluA2 structure [PDB: 3KG2 (Sobolevsky et al., 2009)], IgG (Clark, 1997), GFP [PDB: 1GFL (Yang et al., 1996)], and Cy3 are identical. Each method for delivery of a contrast agent and detection has advantages and potential confounds that must be considered before embarking on experimental design, investment, and execution.

With all of the upsides of the genetic and antibody methods, there are some potential pitfalls and caveats that the researcher must be aware of (Figure 1). For instance, with fusion proteins and transfection, it is difficult to specifically control the expression level of the fusion protein. This can sometimes result in confounded results, especially in cases where the native protein is normally tightly controlled by the cell. I would postulate that more examples of this confound have been detected, but not reported due to the nature of the null result. In the neuroscience community, specific examples of this problem have been observed in the fusion expression of certain ion channels including the AMPA subtype of glutamate receptors. The delicate balance of expression level and subcellular location is likely one of the most important functions in maintaining communication between two connected neurons. The interplay of synaptic scaling, both to enhance and diminish communication, is essential in both the developing and developed nervous system (Jackson et al., 2011; Bats et al., 2012; Opazo et al., 2012; Lambo and Turrigiano, 2013). Further, the insertion of the fluorescent protein within a native protein can be difficult without severe perturbations of the protein and/or proper protein targeting, especially in mammalian systems (Baker et al., 2007). More importantly, overexpression of tagged-monomers of protein receptor subunits has been observed to bias receptor subtype composition thus confounding experimental results (Bredt and Nicoll, 2003). However, these issues can be circumvented in genetically-tractable organisms by performing knock-out/knock-in genetics so that the native levels of the protein are maintained as close as possible to the wild-type state.

The antibody method of labeling native proteins, by contrast, many times requires the addition of large multivalent molecules as a primary antibody and then multiple molecules of a similarly-sized secondary antibody to track the movement of receptors. While this method offers the benefit of exquisite specificity due to the multivalent and immunologically optimized interaction with the target, the large size of typical primary antibodies in addition to the size of typical secondary antibodies may impose unforeseen consequences when one would like to study *natural* protein movements on the cell surface. Additionally, when studying proteins that may move into or out of the synapse, the antibody method may be size-limited as to what it can tell us regarding natural movements of receptors and their movement into or out of the synapse (Dahan et al., 2003).

## A compromise between genetics and size

Small affinity tags that specifically bind a fluorescent probe, sometimes as small as a single cysteine amino acid, can be used to covalently attach a fluorophore to a receptor (Griffin et al., 1998; Marks et al., 2004; McCann et al., 2005). In addition, new gene editing strategies (Sun and Zhao, 2013) hold great promise for installing either short sequence motifs that can be recognized by an exogenously applied chemical entity (e.g., FlAsH, ReAsH, or RhoBo Adams and Tsien, 2008; Halo et al., 2009) or for the directed mutation of underlying sequences that can be modified to produce consensus sequences for a variety of post-translational modifying enzymes (Lin and Wang, 2008).

## A different solution

A number of academic laboratories have been developing ligand-targeted delivery for contrast enhancement cargo (Cha et al., 2005; Vytla et al., 2011; Ishida et al., 2013). At the core of these strategies is the ligand which directs the cargo to the right place. By employing medicinal chemistry literature and fundamental knowledge of binding energies, tethered pharmacophores are being developed to allow for the ligand to direct the sometimes-covalent probe to the target protein or receptor. This is where specificity becomes difficult at times. Because targeting of the receptor is reliant on the ligand, the best-case scenario is one in which the tethered ligand only binds to the intended target. In reality, many pharmacophores are believed to be specific at a certain concentration, but off-target competitive and allosteric sites are always rife in the milieu of a cell or tissue. These are in addition to random collisions that can result in covalent modification. Methods to test the specificity of a ligand-directed probe include competitive blockage of labeling, use of orthogonal readouts (biochemical activity, electrophysiology, binding interaction reporters, etc.). In the end, however, it is difficult, if not impossible, to ever definitively determine that a new probe molecule is 100% specific for a desired target. While we can screen the known off-site interactions, the real problem is that there are likely unknown unknowns lurking on the cells and tissue that result in off-target labeling.

One strategy that we have been developing allows for non-invasive modification of protein receptors with a small molecular weight fluorophore for imaging experiments. The strategy may be likened to a molecular-scale bur that secretly sticks to a receptor and provides a read-out of receptor location. Our probes harbor a ligand (agonist, antagonist, or any other ligand that maintains affinity for the target protein) for targeting specificity, a fluorescent dye for optical monitoring of the receptor, and a promiscuous electrophilic group for covalent coupling to the target receptor. In addition, we have also engineered the probe system so that the ligand can be excised from the coupled system via simple and efficient photolysis of the structural core (Vytla et al.). After photolysis, the coupled fluorescent probe/receptor complex consists of a non-liganded receptor that has a small fluorescent probe stuck to its side. This allows for time-resolved tracking of endogenous receptors that can continue to function in their native environment and respond to endogenous ligands and then signal normally. This has allowed us to now track labeled receptors on live neurons and monitor their movements in response to stimuli. In order to track receptor molecules in their native state, it is important to relieve persistently-agonized or antagonized receptors and avoid changes to the receptor that are accompanied by ligand binding. Agonist-induced conformational changes of some receptors, such as the glutamate receptors, have been shown to induce clathrin-mediated endocytosis of the receptors or differential trafficking of these receptors (Nong et al., 2004; Malinow et al., 2005; Dhami and Ferguson, 2006). In addition to agonist-induced changes, antagonist-induced alterations in receptor trafficking have also been described (Carroll et al., 2001).

## Desirable properties for delivery of contrast agents to proteins in cells:

### Traceless

The protein target, the cell, and the observer should detect no difference between the unlabeled (native state) and the labeled form of the target protein. Depending on the protein target, evidence of lack of perturbation may be difficult to prove.

### Specific

The delivery of the contrast agent to the protein of interest should be as specific as possible. Off target labeling will confound downstream results and should be avoided at all costs.

### High contrast

The contrast agent that is delivered should be easy to detect from background signal. For instance, a high quantum efficiency fluorophore should be employed in the case of fluorescence microscopy.

## Long term prospects for traceless labeling

Traceless delivery of contrast agents to endogenous proteins is the logical next step in garnering an unperturbed view of the routines of proteins in live cells. We, and others, continue to develop new pharmacophores to enable ligand-directed labeling of endogenous proteins. During the next few years, it will be interesting to compare chemically-enabled traceless labeling with the other two main methods of protein detection; fluorescent fusion protein expression and antibody-based tracking. I suspect there will always be a trade off between availability of reagents, ease of use, and financial considerations that will dictate which of these now three methods one will choose for a given experiment.
